# Retraction Note: Genistein inhibits stemness of SKOV3 cells induced by macrophages co-cultured with ovarian cancer stem-like cells through IL-8/STAT3 axis

**DOI:** 10.1186/s13046-023-02603-6

**Published:** 2023-01-24

**Authors:** Yingxia Ning, Weifeng Feng, Xiaocheng Cao, Kaiqun Ren, Meifang Quan, A. Chen, Chang Xu, Yebei Qiu, Jianguo Cao, Xiang Li, Xin Luo

**Affiliations:** 1 Department of Gynaecology and Obstetrics, The First Affiliated Hospital of Guangzhou Medical University, Guangzhou, 510120 China; 2 The First Affiliated Hospital of Jinan University, Guangzhou, 510632 China; 3 Department of Pharmaceutical Science, Medical College, Hunan Normal University, Changsha, 410013 China; 4 Key Laboratory of Study and Discover of Small Targeted Molecules of Hunan Province, Changsha, 410013 China; 5 Department of preclinical medicine, Medical College, Hunan Normal University, Changsha, 410013 China

**Retraction Note:***J Exp Clin Cancer Res***38, 19 (2019)**

**https://doi.org/10.1186/s13046-018-1010-1**

The Editor in Chief has retracted this article. Although Fig. 9 was corrected in May 2019 [1] additional concerns have been raised regarding the following issues:

- Figure 2D: the Oct4 blot looks similar to blot described as CD133 in Fig. 7D of [2], which was consideration around the same time and shares two authors with this article ;
- Figure 4C: one of the actin controls looks very similar to the blot previously published [3] in Fig. 4B as a GAPDH control by different authors;
- Figure 7B, the actin control appears similar to the Tubulin one in Fig. 4B in a previously-published paper by different authors [4];
- Figure 8C, the upper actin control seems similar to the a-tubulin blot in Fig. 4B of a previously-published paper [5] by different authors.
- Figure 8C, the lower actin control seems similar to the A549 actin control in Fig. 3A of a previously-published paper [6] by different authors.

Additionally, the tumour sizes in the corrected Fig. 9 do not appear to match the volume bar chart, and the additional tumours in FigS5A appear too large to meet ethical standards around animal welfare.

The authors sent raw photos but were unable to provide the original Western blot images and the ethics approval. The Editor in Chief, therefore, has lost confidence in the integrity of the findings of this article. All authors agree with this retraction.

## References

[1] Ning Y, Feng W, Cao X (2021). Correction to: Genistein inhibits stemness of SKOV3 cells induced by macrophages co-cultured with ovarian cancer stem-like cells through IL-8/STAT3 axis. J Exp Clin Cancer Res.

[2] Wen Q, Xu C, Zhou J (2019). 8-bromo-7-methoxychrysin suppress stemness of SMMC-7721 cells induced by co-culture of liver cancer stem-like cells with hepatic stellate cells. BMC Cancer.

[3] Sui X, Xu Y, Yang J, Fang Y, Lou H, Han W (2014). Use of Metformin Alone Is Not Associated with Survival Outcomes of Colorectal Cancer Cell but AMPK Activator AICAR Sensitizes Anticancer Effect of 5-Fluorouracil through AMPK Activation. PLoS ONE.

[4] McConnell BV, Koto K, Gutierrez-Hartmann A (2011). Nuclear and cytoplasmic LIMK1 enhances human breast cancer progression. Mol Cancer.

[5] Hui W, Yuntao L, Lun L, WenSheng L, ChaoFeng L, HaiYong H (2013). MicroRNA-195 Inhibits the Proliferation of Human Glioma Cells by Directly Targeting Cyclin D1 and Cyclin E1. PLoS ONE.

[6] Choi KE, Hwang CJ, Gu SM, Park MH, Kim JH, Park JH, Ahn YJ, Kim JY, Song MJ, Song HS, Han S-B, Hong JT (2014). Cancer Cell Growth Inhibitory Effect of Bee Venom via Increase of Death Receptor 3 Expression and Inactivation of NF-kappa B in NSCLC Cells. Toxins.

